# Case report: palmar herpetic whitlow and forearm lymphangitis in a 10-year-old female

**DOI:** 10.1186/s12887-019-1828-5

**Published:** 2019-11-21

**Authors:** Leora Lieberman, Daniel Castro, Avni Bhatt, Fred Guyer

**Affiliations:** 1 Department of Pediatrics, Children’s Hospital of Philadelphia, Philadelphia, PA USA; 2 Department of Pediatrics, College of Medicine, University of Florida, PO Box 100296, Gainesville, FL 32610 USA

**Keywords:** HSV, Herpetic whitlow, Lymphangitis, Palmar lesion, Vesicular lesion

## Abstract

**Background:**

Herpetic whitlow is a viral infection caused by the herpes simplex virus (HSV) types 1 or 2, and occurs in the pediatric population primarily on the fingers and toes due to autoinoculation from oral secretions. Because of this cited prevalence, other locations of herpetic whitlow may go unrecognized.

**Case presentation:**

We present an atypical presentation of palmar herpetic whitlow with delayed recognition and associated viral lymphangitis. The patient presented as a transfer from an outside hospital with a progressive, three-day history of a suspected left hand abscess preceded by left hand pain and itching. She was initially evaluated by Orthopedic Surgery, who described an erythematous, edematous, tender, left palmar abscess with associated erythematous streaking up her forearm. The lesion was surgically managed with an incision and drainage. Wound cultures were obtained during which “minimal drainage” was noted. After admission to the General Pediatrics Hospital service, the lesion was noted to appear vesicular and subsequently obtained PCR samples were positive for HSV type 1, confirming her diagnosis of herpetic whitlow. Although she remained afebrile with negative wound cultures throughout her hospitalization, a secondary bacterial infection could not be conclusively excluded due to the accompanying lymphangitis. Thus, she was discharged with oral antibiotics and anticipatory guidance of potential recurrence of palmar lesions.

**Conclusions:**

Herpetic whitlow should be included in the differential diagnosis of palmar lesions that appear vesicular or abscess-like to ensure appropriate treatment. Additionally, these palmar lesions may present with associated lymphangitis without evidence of bacterial infection.

## Background

Herpetic whitlow is a painful cutaneous infection caused by the herpes simplex virus (HSV) types 1 or 2, and occurs in the pediatric population primarily due to autoinoculation from oral or genital secretions. Classic presentations of infection include a deep-seated, swollen, erythematous, and vesico-ulcerative lesion of the fingers that may be preceded by prodromal numbness, tingling, burning, pain, or itching in the affected location [1–3]. Associated symptoms can include fever, lymphangitis, and regional lymphadenopathy [2]. Pharmacological therapy is usually not necessary as the course is self-limiting. Herpetic whitlow can be misdiagnosed as a bacterial infection resulting in unnecessary incision and drainage, as vesicles may be slow to develop or not develop at all [2].

Here, we present a case of herpetic whitlow on the palm with delayed recognition and associated forearm lymphangitis. The delay in diagnosis was likely due to the cited prevalence of cases on fingers and toes [4–7]. In 1992, one previously reported case of pediatric herpetic whitlow was found to be a primary palmar infection as seroconversion was documented [8]. However, autoinoculation may also occur on any part of the patient’s extremities, as demonstrated in this report. Lack of knowledge of alternate locations may lead to delayed diagnosis, incorrect management, and subsequent complications. Our report highlights an atypical presentation of palmar herpetic whitlow with forearm lymphangitis in an HSV type 1 positive child.

## Case presentation

**Fig. 1:**
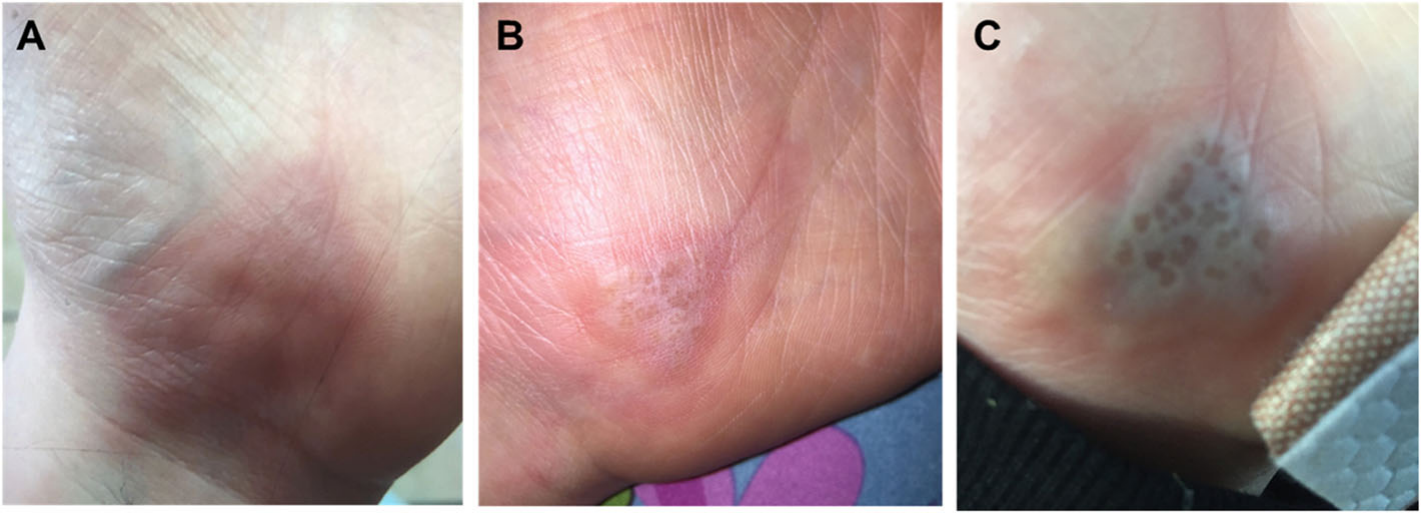
Left hand lesion **a**) 3 days prior to presentation **b**) 1 day prior to presentation and **c**) on the day of presentation

A ten-year-old female initially presented to our Emergency Department as a transfer from an outside hospital with a suspected left hand abscess. Three days prior to presentation, the patient cited 9/10 pain and itching on her left palm that had subsequently progressed to an overlying erythematous lesion (Fig. 1a, b). Oral Tylenol and topical hydrocortisone cream provided no relief at home, and thus they presented to their local community hospital for treatment. On presentation at the outside hospital, she had a white blood count of 8900/mm^3^ with differential (64.5% neutrophils, 19.5% lymphocyte, 12.1% monocytes, 3.1% eosinophil, 0.8% basophil). She was started on intravenous clindamycin and transferred to our hospital. There was no history of recent trauma to her left palm, but her mother did note that she would sometimes suck and bite on the base of her palm. There was no history of oral, fingertip or toe lesions. She had no significant medical history, no current medications, no known allergies, no developmental delay, and no family history of methicillin-resistant or susceptible *Staphylococcus aureus* (MRSA, MSSA) skin infections or herpetic lesions. Four years prior to presentation, she had experienced a similar lesion on the same palmar location that was treated surgically with incision and drainage followed by an unknown course of antibiotics.

**Fig. 2:**
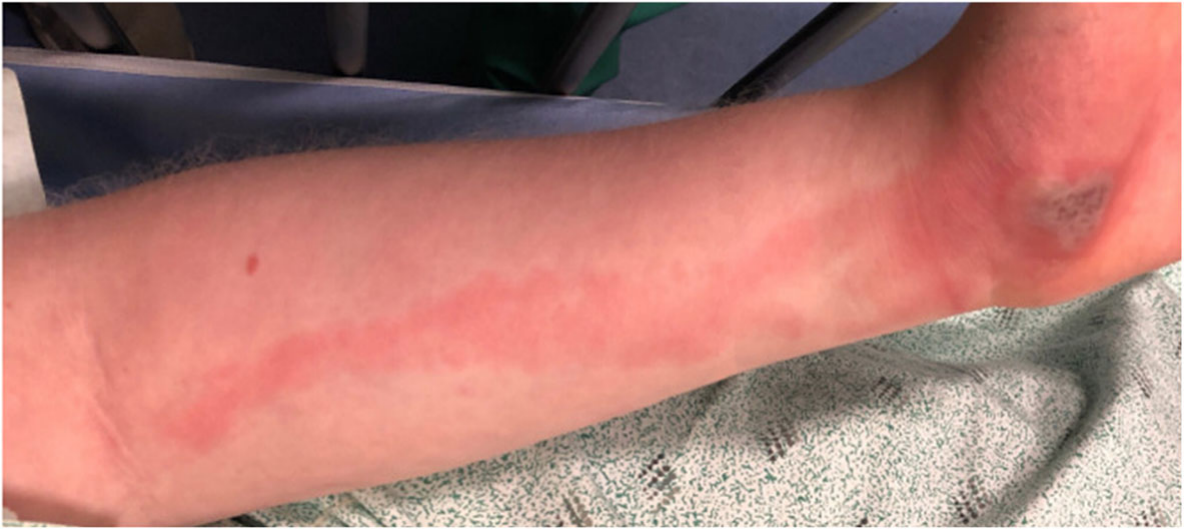
Left forearm erythematous streaking

After transfer to our institution, the patient was initially evaluated by Orthopedic Surgery, who described an erythematous, edematous, tender left palmar abscess with associated erythematous streaking up her forearm. There was no numbness, tingling, fever, cough, dyspnea, abdominal pain, nausea or vomiting. She was afebrile, hemodynamically stable with normal mental status. Her left upper extremity had full range of motion and appropriate strength, with no lymphadenopathy, and radial pulses intact. Based upon these findings, no additional laboratory tests were performed. X-rays of the left forearm and hand showed soft tissue edema with no acute osseous abnormalities. The lesion was surgically managed with an incision and drainage, during which “minimal drainage” was noted. Wound cultures were also obtained. After admission to the General Pediatrics hospitalist service, the lesion was noted to appear vesicular from photos taken prior to surgical management (Fig. 1c). Thus, HSV infection was suspected and PCR samples were obtained from an unroofed left palmar vesicle. These samples confirmed the presence of HSV type 1, resulting in her diagnosis of herpetic whitlow. The initial wound cultures had no growth after 5 days, further confirming that the palmar lesion was not due to a bacterial infection. However, the associated erythematous streaking up her forearm was identified as lymphangitis, indicating a possible bacterial infection secondary to HSV (Fig. 2). Although the absence of fever reduced suspicion for bacterial infection, she was discharged on a 7 day course of oral clindamycin. A secondary bacterial infection could not be conclusively excluded as she had improvement in the lymphangitis while on clindamycin. She was advised to follow-up with her pediatrician within 1–2 days of discharge and discuss the option of acyclovir prophylaxis to prevent recurrence if the lesions occur more frequently moving forward. At that time, it was not recommended that the patient consider prophylaxis, as the two documented occurrences happened 4 years apart. As of 1 year post discharge, she has not returned to our institution for related conditions.

## Discussion and conclusion

Here, we presented a case of a left, palmar lesion caused by herpetic whitlow that was initially mistaken for a left palmar abscess caused by MSSA or MRSA. Classically, herpes infection of the hand in children is associated with finger sucking in asymptomatic salivary carriers [9]. However, viral vesicles can involve any region of the hand. While reviewing her history, certain points hint to this eventual diagnosis, including the prodromal symptoms (pain and itching in the affected area), the history of a previous occurrence, the palm sucking and biting, the lack of leukocytosis, and the lack of any significant or purulent drainage. The palm sucking and biting behavior is unusual in a 10 year-old child, indicating that the patient may have had a behavioral disorder that contributed to this habit. However, no documented evaluation was present in the patient’s chart. Our diagnosis was confirmed by PCR, but our results were complicated by the fact that antibiotics were started at the outside hospital prior to the drawing of wound cultures.

Although the initial diagnosis was a bacterial abscess secondary to MSSA or MRSA, other causes of palmar lesions include herpetic whitlow (as in this case), cellulitis, coxsackie viruses, erythema multiforme, sporotrichosis, syphilis, rat bite fever, impetigo, or trauma. In cases of herpetic whitlow, incision and drainage is contraindicated for risk of viremia, secondary bacterial superinfection, and HSV encephalitis [7, 10, 11]. While our patient did not experience any of these secondary complications, it is important to carefully consider a broad differential diagnosis prior to surgical management. For consideration to begin acyclovir prophylaxis, several recurrent episodes within 1 year would need to occur. Since the patient had only two reported occurrences of HSV type 1 lesions, prophylaxis was not indicated at the time. However, confirming the diagnosis of herpetic whitlow allowed us to provide adequate anticipatory guidance to the patient regarding the recurrence of these lesions and awareness of the prodromal phase during which treatment can be initiated.

Our case was further complicated in that our patient had an associated viral lymphangitis, a rarely reported complication of HSV [12–14]. The presence of associated lymphangitis raised initial concerns for a bacterial infection. Our report aims to document a case in which palmar lesions in combination with lymphangitis broadened the initial differential, but was ultimately an atypical presentation of HSV type 1 herpetic whitlow to be considered for future cases.

## Data Availability

Not applicable.

## Abbreviations

HSV: Herpes Simplex Virus
MRSA: Methicillin-resistant *Staphylococcus aureus*
MSSA: Methicillin-susceptible *Staphylococcus aureus*
PCR: Polymerase Chain Reaction
